# Cases of Scarlatina Anginosa, Treated by Mr. R. Hamilton and His Nephew

**Published:** 1811-02

**Authors:** 


### *To the Editors of the Medical and Physical Journal.*

### *Cases of Scarlatina Anginosa, treated by* Mr. R. Hamilton *and his* Nephew.

Gentlemen,

FINDING some remarks and cases of this formidable and fatal disease, in your last Number, by Mr. Goodwin, who as well as you, solicit farther particulars on this head, I take the liberty to transmit, for your consideration, some cases and remarks on this interesting subject.

Before proceeding further, I beg leave to observe, that the disease has raged here since September 1809, during which it has assumed many anomalous appearances both in its duration and symptoms, from the slightest affection to the most destructive malady. Most authors since the time of Sydenham, Pringle, Sims, Fothergill, have considered this disease as highly putrid almost under every form, and prepossessed with this idea, they have had recourse immediately and indiscriminately to tonics and antiseptics.

This treatment seems, however, to be fast losing ground since the publication of Currie's Medical Reports, and Hamilton's Observations on Purgative Medicines. Without entering at present further into a history of the disease, I shall proceed briefly to mention the remedies which we chiefly employ. These are bleeding, blistering, vomiting, purging, cold affusion, sponging, &c. with the strictest antiphlogistic regimen.

These powerful means, it must be understood, are not to be used indiscriminately, for in many instances, that early stage, in which their use would be attended with the greatest success, has elapsed before the medical practitioners can arrive. In the last stage only, are tonics and antiseptics admissible, and even here, when the throat is much affected, I have seen much more beneficial results from fomentations and repeated poultices, applied externally after blistering the neck, with the addition of inhalations of volatile and aromatic vapours. I may just mention that venesection was adopted chiefly from observing epistaxis to be a frequent symptom, and favourable in every case in which it occurred.

*Cases shewing the success resulting from Venesection.*

Case I. Miss Shepherd, aged 14, had been attending her uncle, who died of the disease on the fourth day. She was seized the day after his decease, with cold and hot fits, great pain of the head, throat much affected, pulse quick and strong, eruption appearing. She was directed to lose six ounces of blood, which immediately relieved her head; had a purge, was repeatedly sponged with vinegar and water, drank saline mixture frequently; pursuing this plan for three or four days, she recovered rapidly, and no hydropic symptoms followed.

Case II. Miss Right, aged 8.—This girl had the disease in a violent degree, being delirious. She was bled, sponged, purged; purged; and the throat being very sore, it was blistered, and afterwards repeatedly poulticed. The eruption continued out six days, and appeared in several places in groups of small pustules, which contained matter, &c. She soon recovered, and slight hydropic symptoms followed.

Case III. Miss Denny, aged 14, was taken in the night with vomiting, purging, great pain of the head, much fever, and no appetite, throat sore. Was bled next morning about six ounces, and was sponged. Next morning she had so far recovered as to allow her to sit up, and on the following day returned to her usual occupations. In this case the disease, which attacked violently, was evidently cut short by the active remedies.

Case IV. C. Turner, aged 10. This girl was so bad when first visited, as to render her recovery extremely doubtful. She had much fever, had been delirious, throat so much affected, that she could with the greatest difficulty articulate; tongue covered with a brown crust, eruption very full. This was the second day of the disease. I extracted about six ounces of blood from the arm, gave a purge, applied a blister to the throat, and ordered frequent ablution. In the evening she was much better, took saline mixture frequently, and had the throat repeatedly fomented and poulticed. She recovered so rapidly, that in a few days she became well. She experienced slight rheumatic pains of the joints, but had no swellings.*

Case V. Sarah Cork, aged 8, was attacked with the fever in the usual manner. When visited, the throat was much affected, the eruption very full, considerable pain of the head, no appetite, pulse rapid and full. She was immediately bled about six ounces, took a purging powder, and was ordered to be sponged frequently in the usual manner. The ensuing morning she appeared nearly well, her purge operated, and she slept well during the night. After which she recovered very fast, and was no longer confined to her bed.

Case VI. Mr. Tydiman took the disease from his own children, five of whom laboured under it at the same time. Throat sore, deglutition difficult, tonsils inflamed, pains of back and loins with cold chills, and much pain of the head, with sickness. His tonsils were scarified and bled freely; he took an emetic, and afterwards a purge. Next day he was

* N. B. Three children out of five died next door, all of whom, the mother informed me, had been well plied with bark and wine from the commencement of the disease.

somewhat somewhat better, but the pain of the head had little abated, and the fever was considerable. He was now bled to the quantity of sixteen ounces, took saline mixture frequently, and occasionally gargled his throat. Next morning he was much better, and the pain of the head had quite subsided. After this he recovered very fast, returning to his work on the third day after.

Case VII. Miss Tydiman, aged 12, was attacked in the usual way; took a purge which operated well; after which she became very sick and vomited repeatedly. Fever considerable, great prostration of strength, eruption out. She was now bled about eight ounces; immediately after which she again vomited. The stomach remained so irritable, that nothing could be retained; an opium plaister was therefore applied to her breast, which gave her a good night's rest, and she awoke next morning nearly free from fever, and became quite well in a few days.

Case VIII. Godbold, aged 7, was seized with the disease on the recovery of her sister. She complained of much pain of the head and great difficulty in swallowing, eruption full, had been delirious the two last nights. She lost between five and six ounces of blood, which gave immediate relief to the head, and no more delirium occurred. In a day or two after she was quite well. The cuticle was thrown off in very large portions, but no anasarca succeeded.

Case IX. Mrs. Southerfield caught the disease from her infant child, who died of it soon after her attack. Her throat was much affected, great pain in the head, much apparent debility. She was bled about twelve ounces, purged, and had a blister applied to the throat, and frequently used Mudge's inhaler. The common saline mixture was frequently exhibited. Notwithstanding the most serious apprehensions were entertained concerning her recovery, her mind being much affected in consequence of her child's death, as well as her bodily disease, yet she soon recovered her pristine vigour.

### *Cases indicative of the beneficial Results of this Treatment at an advanced Period of the Disease.*

Case I. William Brown, ætat. 10, was attacked on the 20th July. The eruption appeared on the following day, and gradually became worse till the 24th, when first visited. At this time the throat was much affected, tongue and lips brown, great pain of the head; had been delirious during the night, seemed stupid and comatose, no appetite, great thirst, body costive, pulse quick, talking often incoherently. In this this desperate state I immediately bled him about six ounces, gave him a purge, and ordered saline mixture. He was also repeatedly sponged, and warm fomentations were frequently applied to the throat. Next morning he was rather better; pain of the head had subsided, and he had not been delirious during the night; had had no stool; the purge was therefore repeated; and as he now refused every kind of medicine, he was allowed toast and water only, the sponging and poulticing being continued. He had a motion towards evening. His neck, breast, and arms began now to vesicate, and on the following day these parts seemed as if they had been covered with cantharides. These vesicles began to disappear about the 8th day of the eruption, when the cuticle fell off in large pieces; after which he soon became convalescent, and no dropsical symptoms appeared.

Case II. ——— Downs, ætat. 11. This boy had been ill for several days before I saw him. He now complained of great pain of the head, and sore throat, (the scarlet eruption had appeared and disappeared several times during the last three days) anorexia, and considerable fever. I bled him about seven ounces, which gave the head great relief. He also took cooling medicine. On calling the next day, he was free from all complaint, and had gone out of doors.

Case III. F. Web, ætat. 14, was attacked with the disease some days before I visited her. Her fever was very high, body covered with the eruption, throat much affected, bowels open. Bleeding was now proposed, but not acceded to; she was therefore sponged as usual, took saline mixture plentifully, and had the throat poulticed. On the fifth day of the disease she became much worse, being quite delirious and rather comatose, the eruption still out, very full, and great pain of the head, &c. In this state she was bled about seven ounces, after which she became much better, and the delirium did not again recur. The ablution and medicine were continued for a few days, when she became convalescent. Slight œdema succeeded.*

Case IV. Maria Peak, ætat. 15, was seized on the 6th of September. The following day she took an emetic and purge which operated well. She gradually became worse till the ninth, when she was bled, sponged, &c. At this time the eruption was very full, the throat considerably swelled, scarcely admitting any drink, great pain of the head. Next morning she was very little better, and towards evening ap-

* N. B. This girl remained for several days in the utmost danger.

peared to be sinking fast. She was now ordered a cordial and stimulating draught. This evidently increased every symptom, and was not, therefore, repeated. She was now kept to the strictest antiphlogistic remedies. The neck was blistered and afterwards repeatedly poulticed, and the inhaler frequently used. Epistaxis occurred towards night. This and the copious discharge of saliva seemed the only favourable symptoms. The putrid smell was now almost insupportable. Nothing could be swallowed for the two last days. Our remedies were all therefore external, viz. fomentations, blisters, inhalations, and ablutions, &c. By these means only was she, at length, rescued from the arms of death. Her convalescence was, however, much retarded by a large abscess formed on the neck. This was one of the most desperate cases conceivable, she being for ten days almost in articulo mortis, during which time the putrid odour could scarcely be kept under by continued fumigations, &c.

Case V. Sarah Barrel, ætat. 15. The only material difference in this case from the last, was, that she remained quite delirious for seven days, being often with great difficulty retained in bed. The head had been thrice blistered, and sinapisms were applied to the feet. Neither food nor medicine were swallowed for the greater part of the time. She was allowed weak wine and water when convalescent. Epistaxis occurred after the bleeding, and the salivary secretion was copious. No hydropic symptoms succeeded.

*Cases in which Phlebotomy appeared to check the Progress of the Disease when early adopted.*

Charlotte Peak, ætat. 13, sister to the subject of Case IV. was attacked on the 14th of September, and the disease fast advancing, the greatest danger was to be feared. She lost about seven ounces of blood, and took an active purge when the eruption began first to appear. She was delirious on the second night of the attack, but every symptom speedily disappeared after the venesection and operation of the purge, when she was no longer indisposed.

Case II. ——— Manning, ætat. 14, was on a visit, and the fever being in the neighbourhood, she caught the disease. I saw her next morning, when she complained of much pain of the head, sore throat, no appetite, pulse rapid, very thirsty, face flushed. She was now bled about eight ounces, took a purge, was sponged as usual, and ordered saline mixture and barley water. Next day she was nearly well, appetite good, and no particular complaint. In the evening she was so well as to return home.

Case

Case III. Mrs. Collier was attacked with the disease in the usual manner; had great pain of the head, sore throat, and cold shiverings, &c. She was immediately bled between eight and ten ounces, and afterwards purged and sponged, &c. Next morning the body was covered with spots as large as a crown-piece. Towards evening she left her bed, and was quite recovered on the succeeding day.

Case IV. Eliz. Right, ætat. 15, returned from a visit on the 2d of November, when her sister lay bad under the complaint. She was attacked on the 12th with sickness and vomiting, pains of the joints, sore throat, much pain of the head, and great thirst, &c. She was now bled about six ounces, sponged, and took saline medicine. In the evening I was agreeably surprised to find her sitting at the fire, being nearly well. No eruption appeared, the throat better, and the appetite good. Next day she became quite well.

*Cases shewing the comparative Success of each Method of Treatment.*

A family of the name of Porter, residing at Westerfield Green, was attacked with the disease. It consisted of five children, the eldest of whom was about thirteen, and the youngest near two years.

James was seized on Wednesday. I saw him on Thursday evening, when I learned he had taken an emetic, which both purged and vomited him. He was allowed toast and water, and had a blister for the neck. Some days after he began to recover, but did not leave his bed for seven or eight days.

The eldest was taken on Friday night. Saturday morning she took a purge of rhubarb, which also vomited her; she drank freely of toast and water, &c. and in the night became delirious. Sunday morning the fever was very high, and about twelve o'clock she became stupid and comatose. Camphor, aromat. conf.* and wine were ordered, and she continued till towards morning in a similar state, when she expired.

The second boy was seized some days after. He was also purged, and had vomited likewise. The eruption was full, and the throat slightly affected; on the fourth day he became delirious and comatose. He was now ordered camphorated julep, with aromatic confection, and wine. This plan he continued for several days without any apparent benefit. The delirium and stupor continuing, he died on the eleventh

* Local circumstances, not design, prevented the adoption of means here, which might have saved her.

day of the disease. His smell was so bad for some days previous to his decease, as to be almost insupportable.

The two younger children took the disease at the same time, but had it slightly, and were therefore chiefly left to nature. Their convalescence was, however, very tedious.

A family of the name of Barrel, in the parish of Bramford, caught the disease. This family consisted of six children, the eldest aged seventeen, the youngest about one.

Charlotte, aged five, had it first, and was treated with the strictest antiphlogistic regimen, and, notwithstanding she had been delirious, she soon recovered.

At this time the three eldest were taken, viz. Betty, aged seventeen, Sarah, fifteen, and Jane, eleven years. They all lay in a small lower room, very little larger than what the bed occupied. Two, and for the most part three were in the bed, and one on a sofa. They were all seized with sickness, vomiting, and purging. Sarah became delirious, face flushed, eyes vacant, and the adnata covered with blood-vessels, great pain of the head, &c. This was on the second day of the disease. She was now bled about six ounces, had a blister applied to the nape of the neck, was repeatedly sponged, and drank saline mixture and aq. ammon. acetat. The throat being much affected, was continually poulticed. The delirium and stupor continuing, both temples were blistered, and sinapisms applied to the feet. Persisting in this plan for some days, she became convalescent, and was now allowed some wine. No hydropic symptoms succeeded, and she recovered remarkably fast. Betty and Jane continued to vomit and purge for two days, when it was judged prudent to check this by a small dose of opium. They drank saline mixture plentifully, and were repeatedly sponged. Jane began first to recover. Betty became delirious; a large blister was now applied to the nape of the neck and temples. The throat was often fomented, and the inhaler frequently used. By these means the delirium soon subsided, and the eruption beginning to desquamate, she became convalescent. At this period Mary, aged nine years, was seized in the same violent manner, soon becoming delirious. She was actively purged, vomited, sponged, &c. and allowed the saline mixture as usual. Soon after this she recovered. The young child escaped the disease.

*N. B.* These five were all confined in the above small apartment, not only three, but often four lying in the same bed. The disease attacked with the greatest violence, inducing delirium, a wild countenance, and almost complete loss of strength in the course of the second and third day. A respectable medical gentleman in the neighbourhood, having visited visited them, pronounced the approaching dissolution of three. They were attended with the greatest care, and the plan pushed to its utmost extreme.

To each of these heads might be added, a numerous list of examples, but considering these sufficiently indicative of the success of this treatment, I refrain from occupying more of your useful miscellany.

I remain, Sir, Yours, &c.

W. H.*

*Ipswich, December* 5, 1810.

* We are requested to state that this communication, as well as, the case of puerperal Fever in No. 138, the case of diseased Bladder in No. 140, and the Report of the Walcheren Fever, in the last number of our Journal, were written by W. Hamilton, Surgeon, Ipswich, and not by Dr. W. Hamilton. Editor.

